# Variability of Psoriatic Arthritis Impact of Disease questionnaire (PsAID12) thresholds in psoriatic arthritis: data from the ReFlaP study

**DOI:** 10.1093/rheumatology/keaf002

**Published:** 2025-01-03

**Authors:** Clementina López-Medina, Clémence Gorlier, Ana-Maria Orbai, Laura C Coates, Uta Kiltz, Ying-Ying Leung, Penelope Palominos, Juan D Cañete, Rossana Scrivo, Andra Balanescu, Emmanuelle Dernis, Sandra Meisalu, Adeline Ruyssen-Witrand, Martin Soubrier, Sibel Zehra Aydin, Lihi Eder, Inna Gaydukova, Ennio Lubrano, Umut Kalyoncu, Pascal Richette, M Elaine Husni, Josef S Smolen, Maarten de Wit, Laure Gossec

**Affiliations:** Medical and Surgical Sciences Department, University of Cordoba, Reina Sofia Hospital, Maimonides Biomedical Research Institute of Cordoba (IMIBIC), Cordoba, Spain; Sorbonne Université, INSERM, Institut Pierre Louis d'Epidémiologie et de Santé Publique, Paris, France; Sorbonne Université, INSERM, Institut Pierre Louis d'Epidémiologie et de Santé Publique, Paris, France; Rheumatology Department, AP-HP, Pitié-Salpêtrière Hospital, Paris, France; Division of Rheumatology, Johns Hopkins University School of Medicine, Baltimore, MD, USA; Nuffield Department of Orthopaedics, Rheumatology and Musculoskeletal Sciences, University of Oxford, Oxford, UK; Rheumazentrum Ruhrgebiet, Herne, Germany; Ruhr-Universität Bochum, Bochum, Germany; Singapore General Hospital, Duke-NUS Medical School, Singapore, Singapore; Rheumatology, Hospital de Clinicas de Porto Alegre, Porto Alegre, Brazil; Rheumatology Department, Hospital Clínic and IDIBAPS, Barcelona, Spain; Rheumatology Unit, Department of Clinical Internal, Anesthesiological and Cardiovascular Sciences, Sapienza Università di Roma, Rome, Italy; Internal Medicine and Rheumatology Departments, Sf Maria Hospital, University of Medicine and Pharmacy Carol Davila, Bucharest, Romania; Rheumatology Department, Le Mans Central Hospital, Le Mans, France; Rheumatology Department, East-Tallinn Central Hospital, Tallinn, Estonia; Rheumatology Unit, Toulouse University Hospital, CIC1463, Inserm, Université Paul Sabatier Toulouse III, Toulouse, France; Rheumatology Department, Gabriel Montpied Hospital, Clermont Ferrand, France; Division of Rheumatology, The Ottawa Hospital Research Institute, University of Ottawa, Ottawa, Canada; Division of Rheumatology, Women’s College Hospital, University of Toronto, Toronto, ON, Canada; Rheumatology Department, North-Western State Medical University, St Petersburg, Russia; Academic Rheumatology Unit, Dipartimento di Medicina e Scienze della Salute ‘Vincenzo Tiberio’, University of Molise, Campobasso, Italy; Department of Internal Medicine, Division of Rheumatology, Hacettepe University Faculty of Medicine, Ankara, Turkey; Service de Rhumatologie, Hopital Lariboisiere Centre Viggo Petersen, Paris, France; Université Paris Diderot UFR de Medicine, Inserm UMR1132 Bioscar, Paris, France; Department of Rheumatic and Immunologic Diseases, Cleveland Clinic, Cleveland, OH, USA; Division of Rheumatology, Department of Medicine 3, Medical University of Vienna, Vienna, Austria; Patient Research Partner, Amsterdam, Netherlands; Sorbonne Université, INSERM, Institut Pierre Louis d'Epidémiologie et de Santé Publique, Paris, France; Rheumatology Department, AP-HP, Pitié-Salpêtrière Hospital, Paris, France

**Keywords:** psoriatic arthritis, patient reported outcomes, PsAID12, remission, disease activity

## Abstract

**Objective:**

To explore thresholds for the Psoriatic Arthritis (PsA) Impact of Disease questionnaire (PsAID12) score against disease activity measures in an observational setting, in patients with PsA.

**Methods:**

The baseline data from the ReFlaP observational, prospective, multicentre and international study were used (NCT03119805). Cutoffs for PsAID12 were determined against disease activity scores, defining disease impact states (i.e. remission, low impact, moderate impact and high impact). Statistics used to assess the optimal cutoff point included Youden’s index and the 75th percentile method, with external anchors (i.e. Disease Activity index for Psoriatic Arthritis [DAPSA], very low disease activity [VLDA]/minimal disease activity [MDA] and single questions for both patients and physicians) serving as gold standards. The diagnostic performance of these cutoffs was evaluated using receiver operating characteristic (ROC) curve analyses.

**Results:**

A total of 410 patients were analysed. Mean (s.d.) PsAID12 score was 3.4 (2.5). The prevalence of remission varied between 12.4% and 36.1%, while low disease activity ranged from 37.8% to 59.8%. PsAID12 performed well against external anchors, with high areas under the ROC curves ranging from 0.75 to 0.94. Using the DAPSA as external anchor, the proposed PsAID12 cutoffs were <1.7 for remission, ≥1.7 to ≤3.1 for low impact, >3.1 to <4.8 for moderate impact and ≥4.8 for high impact. Compared with composite scores, patient and physician opinions performed less stringently.

**Conclusion:**

This study established cutoffs for PsAID12 in a clinical practice observational population, corresponding to remission and varying levels of disease impact. However, these proposed cutoffs need further validation, and an expert consensus is essential to confirm the most accurate thresholds for future use.

Rheumatology key messages

- Thresholds for the Psoriatic Arthritis Impact of Disease questionnaire (PsAID12) have been defined.
- These thresholds varied depending on the external anchor used.
- The proposed cutoffs are <1.7 for remission, ≥1.7 to ≤3.1 for low impact, >3.1 to <4.8 for moderate impact and ≥4.8 for high impact.

## Introduction

Psoriatic arthritis (PsA) is a multifaceted inflammatory disease characterized by significant heterogeneity, contributing to a wide spectrum of alterations in health-related quality of life [1]. The Psoriatic Arthritis Impact of Disease questionnaire (PsAID12) is a robust instrument designed to evaluate patient-important symptoms and the overall impact of PsA [2]. Encompassing 12 distinct health domains, the PsAID12 score effectively measures the multifaceted repercussions of PsA, ranging from physical to psychological aspects. It has been provisionally recommended as a core outcome measure for the health-related quality of life domain of PsA by OMERACT [3, 4].

Thresholds for continuous scores help clinicians interpret the degree of symptoms. A more precise understanding by quantifying impact levels that may correlate with different disease states is crucial. However, thresholds depend on the gold standard or reference measure (anchor) being used.

To date, three threshold proposals have been established for PsAID12. The first defined a value below 4 as representing a patient-acceptable symptom state (PASS) [5]. The other two proposals utilized disease activity as an external anchor to determine PsAID12 thresholds based on the clinical Disease Activity index for Psoriatic Arthritis (DAPSA). One set of thresholds, published by Di Carlo *et al.*, proposed cut-off values for PsAID12 corresponding to different levels of disease impact [6], although this study was limited by a small sample size. Recently, new thresholds for PsAID12 have been calculated in a randomized control trial (RCT) population [7]. However, the suitability of using a different external anchor such as the patient’s or physician’s opinion remains uncertain [8].

The objective of this study was to identify thresholds for PsAID12 in an observational setting, corresponding to degrees (states) of disease impact (i.e. remission, low, moderate and high impact) in PsA, using different reference measures. This was achieved through an anchor-driven approach using several reference definitions of disease activity including composite scores and both patient and physician perceptions.

## Methods

### Study population and study design

The ReFlaP (Remission/Flare in PsA) study has been described elsewhere [9]. Briefly, this was an observational, prospective, multicentre and international (14 countries) study conducted between June 2017 and August 2018 (NCT03119805). Patients were evaluated twice, but only baseline data are used here.

Consecutive adults with a definite diagnosis of PsA according to their rheumatologists, and with >2 years of disease duration were included. All patients provided informed consent, with ethical committee approval at each site.

### Data collection

#### General data

Patient demographic variables (age, gender, work status, level of education), disease characteristics (disease duration, predominant type of PsA: peripheral, axial or entheseal), and current treatment (conventional synthetic DMARDs [csDMARDs] or biologic DMARDs [bDMARDs]) were collected. All elements necessary to calculate the very low disease activity (VLDA)/minimal disease activity (MDA) [10] and DAPSA scores were also collected [11].

#### PsAID12

PsAID12 assesses the impact of PsA across 12 domains using numerical rating scales (ranging from 0 to 10, where higher results indicate higher impact). Domains include pain, physical function, fatigue, coping and depression, among others. The final PsAID12 score is calculated by combining these domains, yielding a score between 0 and 10 (higher scores indicate a worse condition) [7]. This questionnaire is known for its readability, simplicity, reliability and validity [6].

#### Definitions used as external anchors for disease activity levels

Remission was defined using VLDA, which requires the presence of seven items (tender joint count [TJC] ≤1, swollen joint count [SJC] ≤1, enthesitis count ≤1, patient global assessment [PGA] ≤2/10, pain ≤1.5/10, skin psoriasis [Psoriasis Area Severity Index, PASI ≤1 or body surface area, BSA ≤3% and HAQ-DI ≤0.5] and DAPSA ≤4), along with single questions for both patients and physicians (for patients: ‘At this time, is your psoriatic arthritis in remission, if this means: you feel your disease is as good as gone?’ and for physicians: ‘At this time, is the psoriatic arthritis in remission, if this means: the absence of clinical and laboratory evidence of significant inflammatory disease activity?’) [9].

Low impact was defined using MDA (which requires the presence of at least five of the items previously described for VLDA), DAPSA ≤14 and single questions for both patients and physicians (for patients: ‘At this time, are you in low disease activity, if this means: your disease is in low activity but it’s not as good as gone?’ and for physicians: ‘At this time, is the psoriatic arthritis in low or minimal disease activity?’).

The moderate disease impact anchor was based on DAPSA cut-offs of moderate (>14 and ≤28) disease activity [11].

The high disease impact anchor was also based on DAPSA cut-offs of high (>28) disease activity [11].

### Statistical analysis

All patients with complete data for calculating remission and low impact according to all definitions were included in the analysis. Missing data were not imputed, and only complete cases were analysed. Descriptive statistics were used to summarize baseline demographic and clinical characteristics of the patients. Similar to the approach used in previous cut-off development [7], cutoffs for PsAID12 were determined using Youden’s index on receiver operating characteristic (ROC) curve analyses and the 75th percentile method, with the external anchors serving as gold standards.

All statistical analyses were conducted with R version 3.4.0 (http://r-project.org).

## Results

### Population

Out of 466 patients, 410 complete cases were analysed (Table 1). Overall, 208 (50.7%) patients were male, with a mean (s.d.) age of 52.8 (12.4) years. The mean (s.d.) disease duration was 10.2 (8.3) years, indicating a long-standing disease history. Most patients were receiving DMARDs, with 73.6% on csDMARDs and 72.8% on bDMARDs. Disease activity levels were moderate: the mean (s.d.) TJC was 4.9 (9.8), mean SJC was 2.4 (7.3), mean physician global assessment was 3.1 (2.5) and PGA was 4.1 (2.8). The mean PsAID12 score was 3.4 (2.5).

**Table 1.** Clinical characteristics of the included population

	All (*n* = 410)	REM/LDA by DAPSA (*n* = 229)	REM/LDA by MDA/VLDA (*n* = 155)	REM/LDA by patient’s question (*n* = 245)
Male, *n* (%)	208 (50.7)	137 (60.6)	105 (69.1)	68 (42.2)
Age, mean (s.d.), years	52.8 (12.4)	52.8 (12.7)	52.2 (12.1)	53.6 (12.2)
PsA duration, mean (s.d.), years	10.2 (8.3)	10.9 (8.2)	11.4 (8.4)	10.7 (8.6)
Current smoker, *n* (%)	68 (18.1)	33 (15.7)	19 (13.2)	31 (14.2)
Current csDMARDs, *n* (%)	243 (73.6)	142 (76.3)	85 (70.8)	156 (77.6)
Current bDMARDs, *n* (%)	233 (72.8)	145 (79.7)	96 (82.1)	87 (67.4)
Oral glucocorticoids, *n* (%)	67 (18.6)	26 (12.9)	10 (7.5)	34 (15.7)
BSA of psoriasis ≥5%, *n* (%)	37 (9.2)	13 (5.7)	2 (1.3)	21 (12.9)
Tender entheseal points, LEI, mean (s.d.)	0.6 (1.4)	0.1 (0.4)	0.1 (0.3)	0.3 (0.9)
Tender joints count (0–68), mean (s.d.)	4.9 (9.8)	0.7 (1.1)	0.3 (0.7)	2.9 (7.2)
Swollen joints count (0–66), mean (s.d.)	2.4 (7.3)	0.4 (1.0)	0.4 (0.9)	1.4 (5.2)
Physician’s global assessment of PsA, mean (s.d.)	3.1 (2.5)	1.8 (1.7)	1.3 (1.4)	2.4 (2.1)
Patient’s global assessment of pain (0–10), mean (s.d.)	4.1 (2.8)	2.3 (1.8)	1.9 (1.9)	3.2 (2.4)
Patient’s global assessment of PsA (0–10), mean (s.d.)	4.2 (2.8)	2.5 (1.9)	2.1 (2.0)	3.3 (2.3)
DAPSA, mean (s.d.)	17.0 (17.7)	6.2 (3.9)	5.2 (4.4)	11.8 (14.7)
HAQ (0–3), mean (s.d.)	0.68 (0.68)	0.38 (0.49)	0.16 (0.28)	0.50 (0.57)
PsAID12 (0–10), mean (s.d.)	3.4 (2.5)	2.0 (1.7)	1.4 (1.3)	2.5 (2.1)

bDMARD: biologic DMARD; BSA: body surface area; csDMARD: conventional synthetic DMARD; DAPSA: Disease Activity index for Psoriatic Arthritis; HAQ: Health Assessment Questionnaire; LDA: low disease activity; LEI: Leeds enthesitis index; PsA: psoriatic arthritis; PsAID12: Psoriatic Arthritis Impact of Disease questionnaire; REM: remission; VLDA: very low disease activity.

### Disease activity levels

Frequencies of disease activity states varies by definitions used (Supplementary Table S1, available at *Rheumatology* online). The prevalence of remission varied between 12.4% and 36.1%, while low disease activity ranged from 37.8% to 59.8% (Supplementary Figure S1, available at *Rheumatology* online). Moderate and high disease activity (defined solely on DAPSA cutoffs) were observed in 108 (26.3%) and 73 (17.8%) patients, respectively.

### Cutoffs for PsAID12

PsAID12 performed well against the external anchors, based on high areas under the ROC curves ranging from 0.75 to 0.94 (Table 2). As expected, PsAID12 thresholds varied depending on the external anchor used. PsAID12 cut-offs were more stringent (lower) for achieving the VLDA and MDA criteria compared with DAPSA remission and low disease activity (Table 2).

**Table 2.** Calculated cutoffs for PsAID12 for different disease impact levels in PsA using different external anchors

	Remission			Low impact			Moderate impact		
	AUC	ROC Youden’s index	75th percentile	AUC	ROC Youden’s index	75th percentile	AUC	ROC Youden’s index	75th percentile
DAPSA	0.94	1.7	1.4	0.89	3.1	3.1	0.85	4.8	4.5
VLDA/MDA	0.94	1.4	0.8	0.91	2.0	1.9	—	—	—
Patient’s question	0.77	1.7	2.4	0.83	3.7	3.7	—	—	—
Physician’s question	0.75	1.8	3.0	0.77	4.2	4.1	—	—	—

AUC: area under curve; DAPSA: Disease Activity index for Psoriatic Arthritis; MDA: minimal disease activity; PsA: psoriatic arthritis; PsAID12: Psoriatic Arthritis Impact of Disease questionnaire; ROC: receiver operating characteristic; VLDA: very low disease activity.

- For defining remission, PsAID12 cutoffs found using Youden’s index methodology varied depending on the anchor: from 1.4 using VLDA as the anchor, to 1.8 using the physician’s question. Using the DAPSA, this cut-off was established at 1.7. However, when using the 75th percentile methodology, PsAID12 cutoffs for remission ranged from 0.8 to 3.0 using VLDA and the physician’s question as anchors, respectively.
- PsAID12 cutoffs for defining low disease impact were found to be 2.0 and 4.2, using MDA and the physician’s question, respectively, according to Youden’s index methodology, with very similar results when using the 75th percentile technique. Considering the DAPSA as external anchor, this threshold for low impact was established at 3.1. Nevertheless, the area under the curve (AUC) was greater using the DAPSA and MDA as anchors in comparison with patient’s and physician’s questions.
- Cutoffs for moderate disease impact could only be calculated using the DAPSA as the anchor, with a result of 4.5 and 4.8 according to the 75th percentile and ROC techniques, respectively (Supplementary Table S2, available at *Rheumatology* online).

In general, compared with composite scores, patient and physician opinions performed less stringently (i.e. <1.8 and 1.8–4.2 for remission and low impact, according to the physician’s opinion, and <1.7 and <2.4 according to the patient’s opinion).

## Discussion

This study has enabled us to propose thresholds for the PsAID12 score in an observational setting, corresponding to different levels of disease impact. Thresholds were more stringent when using the DAPSA and VLDA/MDA as external anchors compared with patient and physician opinions, and these thresholds also demonstrated a higher AUC. Compared with the previous PsAID12 thresholds proposed in a RCT setting where the DAPSA was established as the external anchor, the thresholds developed in the ReFlaP cohort were less stringent (<1.7 for remission, ≥1.7 to ≤3.1 for low, >3.1 to <4.8 for moderate, and ≥4.8 for high impact). This difference possibly reflects variations in patient status and expectations in clinical practice.

Remission is a complex concept. From the patient’s perspective, little is known about their perception of remission and about which level of symptoms would be acceptable for them [12]. In rheumatoid arthritis, studies have shown that the three most important domains of patient’s perceived remission are the absence or reduction of pain and fatigue and the improvement or maintenance of independence [13]. In PsA, there are few qualitative data on patient’s assessments of remission, but an international qualitative study is ongoing to answer these questions. Patients with PsA have to deal with both symptoms related to their joint involvement, skin and nail psoriasis and its burden. This leads to a multi-faceted disease from the patient’s perspective, which may lead to heterogeneity in what patients would consider to be remission.

For composite scores, we know the prevalence of remission is much lower when defined by VLDA due its stringency explained by the inclusion of joints, enthesitis and psoriasis, and due to Boolean scoring [14]. In fact, our results showed that thresholds for PsAID12 using VLDA as external anchor were the most stringent in comparison with patient and physician opinions. Consequently, the remission rates observed using VLDA thresholds were lower, indicating that this method sets a higher standard for what is considered remission. One potential explanation for the less stringent performance of the physician and patient opinions could be the subjective nature of their assessments, which may emphasize different aspects of the disease and reflect different expectations. Moreover, sex-related differences in disease perception could partly explain these results. In the ReFlaP cohort, male patients were more frequently in remission or LDA according to composite outcomes, yet less frequently in remission or LDA based on their own opinions compared with females. This highlights the need for further studies to investigate these sex-related factors in the perception of remission.

LDA status in our population showed a wider range of variation (from 59.8% to 37.8% defined by the patient’s perception or MDA, respectively), probably reflecting the inaccuracy and uncertainty around this concept or the inclusion of remission in the LDA status.

In our study, moderate and high impact in PsAID12 were defined using DAPSA as an external anchor, as no other comparator was available. Di Carlo *et al.* also established cutoffs for moderate (between 4.1 and 6.7) and high impact (above 6.7), being less stringent than our findings, probably due to the lower sample size and patient’s characteristics [7].

This study had strengths and weaknesses. Recruitment occurred at tertiary care centres, leading to a high percentage of patients on biologics, which may limit external validity. However, representativeness was ensured through an international large-scale selection of consecutive patients with PsA. Due to the absence of consensus on defining disease activity in PsA, both VLDA/MDA and the DAPSA, commonly used scores and recommended by the Treat-to-Target task force [15], were employed as external anchors. Furthermore, cutoffs corresponding to patient- and physician-perceived remission/low impact were identified using innovative questions. Notably, considerable effort was invested in developing these patient questions, involving patient research partners to ensure face and content validity. It should be acknowledged, however, that these questions may emphasize musculoskeletal concepts more than skin psoriasis concepts. By involving patient research partners, some of these limitations could be mitigated, although a balance between different disease aspects remains a challenge.

There is no consensus on the optimal approach for calculating cutoffs for disease impact. These anchors are meaningful, easy to interpret and closely related to the measured concept, which strengthens the study. Moreover, the obtained cutoffs were reasonably close to those from a previous pilot study by Di Carlo *et al.* [6]. Selecting appropriate external anchors for patient-reported outcomes is challenging due to the risk of circular reasoning. PsA composite measures incorporate patient-reported outcomes such as pain and patient global assessment [10]. However, these measures were developed with limited patient involvement, and cutoffs for remission/low impact were not driven by patient input [16]. The specific single questions developed for the present study are not expected to introduce more circularity with PsAID12 than what is typical for patient-reported outcomes. This is important because discrepancies in disease activity assessment can impact on treatment decisions and shared decision-making, highlighting the need for establishing accurate thresholds to ensure that treatment decisions are based on reliable assessment [17, 18]. Nevertheless, PsAID12 should not replace composite scores in clinical practice.

In conclusion, this study has explored cutoffs for PsAID12 in an observational, clinical practice population, corresponding to different levels of disease impact. The proposed cutoffs are <1.7 for remission, ≥1.7 to ≤3.1 for low impact, >3.1 to <4.8 for moderate impact and ≥4.8 for high impact. However, these proposed cutoffs require validation and comparison with recently proposed thresholds from RCT [7]. Subsequently, expert consensus is needed to determine the most accurate thresholds for future use.

## Supplementary Material

keaf002_Supplementary_Data

## Data Availability

The dataset of the ReFlap study is available upon request from L.G.
