# Supplementary material for: Variability of Psoriatic Arthritis Impact of Disease questionnaire (PsAID12) thresholds in psoriatic arthritis: data from the ReFlaP study
Source: Rheumatology (Oxford). 2025 Jan 3;64(5):3090–4. doi: 10.1093/rheumatology/keaf002 (PMC12048079; doi:10.1093/rheumatology/keaf002)
Supplement: keaf002_Supplementary_Data [file keaf002_supplementary_data.docx]

**Variability of Psoriatic Arthritis Impact of Disease questionnaire (PsAID12) thresholds in psoriatic arthritis: insights from the observational ReFlaP study.**

**Supplementary Figure S1. Prevalence of remission and low disease activity according to different definitions.**

**
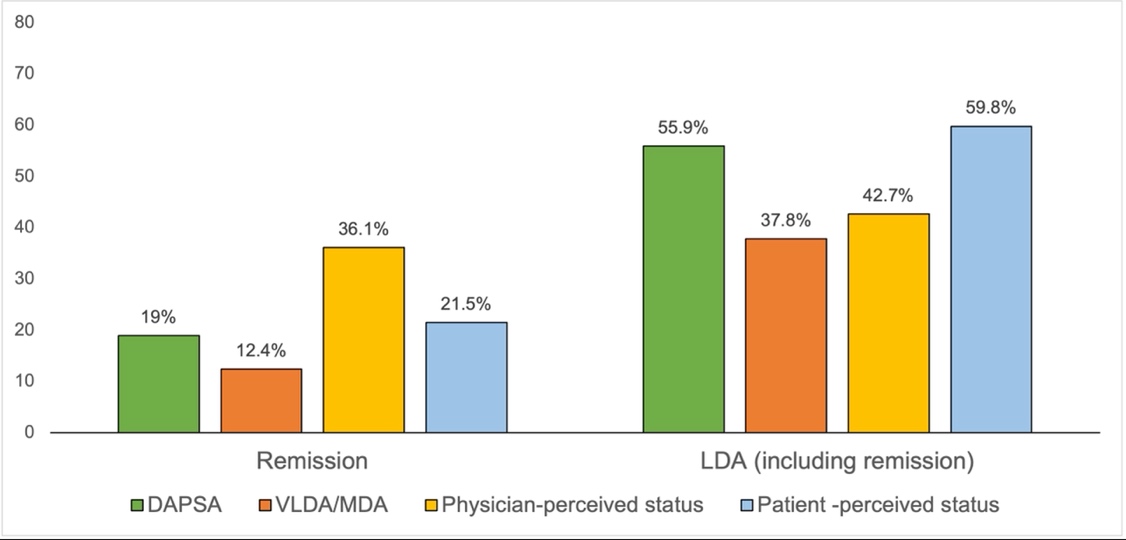
**

DAPSA: Disease Activity index for Psoriatic Arthritis; MDA: Minimal Disease Activity; VLDA: Very low disease activity.

**Supplementary Table S1. Frequency of disease activity states according to different definitions**

|  | **DAPSA** | **VLDA/MDA** | **Physician-perceived status** | **Patient -perceived status** |
| --- | --- | --- | --- | --- |
| **Remission** | 78 (19.0%) | 51 (12.4%) | 148 (36.1%) | 88 (21.5%) |
| **LDA (including remission)** | 229 (55.9%) | 155 (37.8%) | 175 (42.7%) | 245 (59.8%) |
| **Moderate disease activity** | 108 (26.3%) | - | - | - |
| **High disease activity** | 73 (17.8%) | - | - | - |
| DAPSA: Disease Activity Index for Psoriatic Arthritis; VLDA: Very Low Disease Activity; MDA: minimal disease activity; Patient perceived, and physician perceived statuses are based on the single questions for each status. | | | | |
